# Investigation of *Legionella* Contamination in Bath Water Samples by Culture, Amoebic Co-Culture, and Real-Time Quantitative PCR Methods

**DOI:** 10.3390/ijerph121013118

**Published:** 2015-10-19

**Authors:** Akiko Edagawa, Akio Kimura, Takako Kawabuchi-Kurata, Shinichi Adachi, Katsunori Furuhata, Hiroshi Miyamoto

**Affiliations:** 1 Division of Environment Health, Osaka Prefectural Institute of Public Health, Osaka 537-0025, Japan; E-Mails: edagawa@iph.pref.osaka.jp (A.E.); adachi@iph.pref.osaka.jp (S.A.); 2 Division of Microbiology, Department of Pathology and Microbiology, Faculty of Medicine, Saga University, Saga 849-8501, Japan; 3 Division of Planning and Coordination, Osaka Prefectural Institute of Public Health, Osaka 537-0025, Japan; E-Mail: akkimura@iph.pref.osaka.jp; 4 Division of Virology, Osaka Prefectural Institute of Public Health, Osaka 537-0025, Japan; E-Mail: kurata@iph.pref.osaka.jp; 5 School of Life and Environmental Science, Azabu University, Kanagawa 252-5201, Japan; E-Mail: furuhata@azabu-u.ac.jp

**Keywords:** *Legionella*, amoebic co-culture, *Acanthamoeba*, bath water, intracellular growth

## Abstract

We investigated *Legionella* contamination in bath water samples, collected from 68 bathing facilities in Japan, by culture, culture with amoebic co-culture, real-time quantitative PCR (qPCR), and real-time qPCR with amoebic co-culture. Using the conventional culture method, *Legionella pneumophila* was detected in 11 samples (11/68, 16.2%). Contrary to our expectation, the culture method with the amoebic co-culture technique did not increase the detection rate of *Legionella* (4/68, 5.9%). In contrast, a combination of the amoebic co-culture technique followed by qPCR successfully increased the detection rate (57/68, 83.8%) compared with real-time qPCR alone (46/68, 67.6%). Using real-time qPCR after culture with amoebic co-culture, more than 10-fold higher bacterial numbers were observed in 30 samples (30/68, 44.1%) compared with the same samples without co-culture. On the other hand, higher bacterial numbers were not observed after propagation by amoebae in 32 samples (32/68, 47.1%). *Legionella* was not detected in the remaining six samples (6/68, 8.8%), irrespective of the method. These results suggest that application of the amoebic co-culture technique prior to real-time qPCR may be useful for the sensitive detection of *Legionella* from bath water samples. Furthermore, a combination of amoebic co-culture and real-time qPCR might be useful to detect viable and virulent *Legionella* because their ability to invade and multiply within free-living amoebae is considered to correlate with their pathogenicity for humans. This is the first report evaluating the efficacy of the amoebic co-culture technique for detecting *Legionella* in bath water samples.

## 1. Introduction

*Legionella* are gram-negative bacteria and the causative agent of legionellosis, a group of related illnesses that include severe pneumonia and non-pneumonic Pontiac fever [1]. Infection by *Legionella* occurs through the inhalation or aspiration of aerosols generated from contaminated environmental water found in structures such as cooling towers, evaporative condensers of large air-conditioning systems, whirlpool spas, showers, and hot water tanks [1]. Among the *Legionella*, *Legionella pneumophila* (*L. pneumophila*) is the major disease causative agent, although other species such as *L. anisa*, *L. bozemanii*, *L. dumoffii*, *L. gormanii*, and *L. longbeachae* are also human pathogens [1]. More than 1100 cases of legionellosis in Japan, caused by contaminated artificial whirlpool spas or natural hot springs, were reported in Infectious Agents Surveillance Report 2014 [2]. In 2002, a major outbreak originating from a newly opened hot spring spa involved 295 patients with seven deaths [3]. Hence, *Legionella* infection caused by contaminated bath water is an important public health concern in Japan [4,5,6], and sensitive detection and identification of *Legionella* from bath water samples is crucial for legionellosis control.

Culture methods are commonly used to detect *Legionella* in environmental samples, including bath water [7,8,9,10,11]. However, *Legionella* are transformed into a viable but nonculturable (VBNC) form by certain environmental conditions [12,13,14]. Recently, quantitative real-time PCR (real-time qPCR) specific for *Legionella* 16S rRNA, 5S rRNA, or macrophage infectivity potentiator (*mip*) genes have been widely applied to overcome the limitations of standard culture methods [15,16,17,18]. Real-time qPCR is much more sensitive for detecting lower levels of contamination compared with culture methods. Moreover, real-time qPCR can detect nonculturable *Legionella* such as the VBNC types.

In natural aquatic environments, *Legionella* are taken up by FLA including *Acanthamoeba*, *Vahlkamphia,* and *Hartmannella,* by phagocytosis [1]. *Legionella* have the ability to survive and multiply in FLA and are released from FLA into the environment [1]. In addition, *Legionella* can multiply in FLA and recover their culturability [12,19]. To use this association between *Legionella* and FLA for the sensitive detection of *Legionella*, an amoebic co-culture technique was developed to isolate *L. pneumophila* and *L. anisa* from clinical samples [20,21]. In particular, Schalk *et al*. (2012) isolated several *Legionella*, including *L. pneumophila* belonging to sequence types isolated from legionellosis patients in the Netherlands [22]. These reports suggested that the amoebic co-culture technique was useful for detecting *Legionella* responsible for human disease in environmental water samples. However, the efficacy of the amoebic co-culture technique for detection of *Legionella* from bath water samples has not yet been determined.

In this study, we investigated *Legionella* contamination in bath water samples, collected from 68 bathing facilities in Japan, by culture, culture with amoebic co-culture, real-time quantitative PCR (qPCR), and real-time qPCR with amoebic co-culture. 

## 2. Materials and Methods

### 2.1. Sample Collection

Bath water samples were collected from 68 hot spring spas located in Osaka Prefecture, Japan. A total of 68 water samples of 500 mL each were collected in sterile bottles containing sodium thiosulfate at a final concentration of 0.01%. Sample temperature and free chlorine concentration were measured at the time of sampling. Free chlorine concentration was measured by the diethyl-p-phenylenediamine (DPD) method (DPD test Wako; Wako Pure Chemical Industries Ltd, Osaka, Japan). Water samples were immediately delivered to the laboratory at 4 °C, and microbiological analyses were performed on the day of collection.

### 2.2. Sample Processing

Each bath water sample was concentrated by filtration through a 0.22 µm pore-size polycarbonate filter (Advantec Tokyo Co. Ltd., Tokyo, Japan). The membrane was then immersed in 5 mL of sterile deionized water, vortexed for 1 min, and shaken vigorously 50 times. A 3 mL aliquot of the suspension was heated in a water bath at 50 °C for 30 min and used for culture with or without the amoebic co-culture technique as described below. The remaining 2 mL of suspension was stored at −20 °C for DNA extraction.

### 2.3. Isolation and Characterization of Legionella by The Culture Method

Aliquots (100 µL) of the 3 mL treated samples were inoculated onto Wadowsky-Yee-Okuda agar plates containing 5µg/mL vancomycin and 100 U/mL polymyxin with alpha-ketoglutarate (WYO-alpha plates; Eiken Chemical Co. Ltd., Tokyo, Japan). After incubation for 5–7 days at 37 °C, 1–50 colonies showing characteristics of *Legionella* species were selected and cultured on blood agar plates (Nikken Seibutsu Co. Ltd., Tokyo, Japan) and on buffered charcoal-yeast extract agar plates with alpha-ketoglutarate (BCYE-alpha; Eiken Chemical Co. Ltd.). After three days at 37 °C, iolates that grew on BCYE-alpha but did not grow on blood agar were examined by gram staining. Gram negative staining was considered suggestive of the presence of *Legionella* species. The colonies were observed under UV light and 1–5 colonies were randomly selected for the latex agglutination test (Kanto Chemical Co., Tokyo, Japan) and the immune serum agglutination test (Denka Seiken Co. Ltd., Tokyo, Japan) to identify the serogroup of *L. pneumophila*, *L. bozemanii*, *L. dumoffii*, *L. gormanii*, and *L. micdadei*. In addition, DNA-DNA hybridization tests were performed according to the manufacturer’s instructions (Kyokuto Pharmaceutical Industrial Co. Ltd., Tokyo, Japan).

### 2.4. Amoebic Co-Culture Technique

*Acanthamoeba castellanii* strain ATCC 30234 was grown in 75 cm^2^ culture flasks at 30 °C for 4 days with 50 mL of peptone/yeast extract/glucose (PYGC) medium (10 g proteose peptone, 10 g yeast extract, 10.1 g glucose, 5 g NaCl, 0.95 g L-cysteine hydrochloride, 1.74 g Na_2_HPO_4_, and 1.36 g KH_2_PO_4_ in 1 liter of distilled water with the pH adjusted to 6.8). The cells were harvested by centrifugation and re-suspended in PYGC medium to approximately 1 × 10^5^ /mL. The amoebal suspension was distributed into each well of 12-well micro-plates at 30 °C until the cells formed monolayers. Just before processing the water samples, the PYGC medium was removed from each well, and the cells were washed twice with 1 ml of Neff’s amoebae saline (120 mg NaCl, 3 mg MgCl_2_, 3 mg CaCl_2_, 142 mg Na_2_HPO_4_, and 136 mg KH_2_PO_4_ in 1 liter of distilled water).

From the 3 mL processed bath water samples, a 1.1 mL sample solution was inoculated into the amoebal micro-plate wells (amoebic co-culture). After incubating for 5–7 days at 30 °C, 100 µL from the amoebic co-cultures were inoculated onto WYO-alpha plates as described above. DNA was isolated from 1 mL of the amoebic co-culture plates and used for real-time qPCR (68 samples) as described below. When heavy contamination with other bacteria was observed in the culture with amoebic co-culture, DNA extracted from the amoebic co-culture plates, which was stored at −20 °C, was also used for 16S rRNA gene PCR and sequencing to detect the presence of *Legionella* in the samples (14 samples).

### 2.5. DNA Extraction

DNA was extracted using the simplified alkaline DNA preparation method previously described [18]. In brief, 1 mL of a 50-fold concentrated sample was centrifuged at 13,000 g for 10 min at 4 °C, and the supernatant was discarded to a volume of 40 µL. A suspension of the pellet (40 µL) was mixed with 50 µL of 50 mmol/L NaOH by vortexing and then boiled for 15 min. After rapid cooling, the material was neutralized with 8 µL of 1 mol/L Tris-HCl (pH 7.0) and centrifuged at 13,000 g for 10 min at 4 °C. The supernatant was stored at −20 °C until use.

### 2.6. Quantitative Real-Time PCR (Real-Time qPCR)

Real-time qPCR was performed as previously described using a Cycleave PCR *Legionella* (5S rRNA) Detection Kit (Takara Bio Co., Shiga, Japan). Genomic DNA extracted from *L. pneumophila* (ATCC 33152), as described above, was used as the external standard. The number of bacterial cells used for the initial purified DNA solutions were calculated according to the method of Joly *et al.* [16]. PCR reactions with duplicate standards, positive and negative controls, and samples were performed using an ABI PRISM 7900HT Real-time qPCR System (Applied Biosystems, Foster City, CA, USA) according to the manufacturer’s instructions. In brief, reaction mixtures (25 µL) contained 12.5 µL of 2 × Cycleave Reaction Mixture, 5 µL of 5S primers/Probe Mix, 2.5 µL of dH_2_O, and 5 µL of each DNA sample. Cycling conditions were an initial incubation at 95 °C for 10 s, followed by 45 cycles of denaturation at 95 °C for 5 s, annealing at 55 °C for 10 s, and extension at 72 °C for 20 s. The number of cells in each sample was automatically calculated by comparing the threshold cycle values to our constructed standard curve using the ABI PRISM 7900HT SDS Software (Applied Biosystems). It was confirmed that amoebic DNA is not amplified by the real-time qPCR (data not shown). 

### 2.7. 16S rRNA Gene PCR and Nucleotide Sequencing

To examine the presence of *Legionella* in samples that had exhibited bacterial overgrowth (n = 14), a 16S rRNA gene PCR was performed as described previously [18]. PCR product specificity was confirmed by Southern blot hybridization with a 386-mer digoxigenin-labeled *Legionella* 16S rRNA probe. It was confirmed that amoebic DNA is not amplified by the 16S rRNA gene PCR (data not shown). The PCR products were purified using a QIAamp PCR purification kit (Qiagen K.K., Tokyo, Japan) and directly sequenced in both directions using forward and reverse primers with a BigDye Terminator v1.1 Cycle Sequencing Kit (Applied Biosystems). Homology searches were performed using the BLAST software on the NCBI home page (http://www.ncbi.nlm.nih.gov).

## 3. Results 

### 3.1. Legionella-Positive Culture Samples

Using conventional culturing, *Legionella* were detected in 11 of the 68 samples (16.2%). The details of these 11 samples are shown in Table 1. *L. pneumophila* was identified positive in all 11 isolates by the culture method. Among these, serogroups 1 and 5 were predominant. Bacterial numbers ranged from 7.0 × 10^2^ (sample No.#27 and #51) to 1.6 × 10^5^ (# 2) colony-forming units (CFU) /L. In contrast, when using the conventional culture method combined with the amoebic co-culture technique, *L. pneumophila* was detected in only two of the 68 samples (#42 and #51 in Table 1). #51 had a bacterial load of 2.5 × 10^5^ CFU/L, and #42 had too many *Legionella* colonies to count. We could not evaluate the presence of *Legionella* in 14 samples because the plates were overgrown with other bacterial species and *Legionella* colonies could not be identified. To examine the presence of *Legionella* in the 14 amoebic co-cultured samples, 16S rRNA gene PCR and sequencing were performed using DNA samples from these cultures. Two samples were *Legionella*-positive, and the remaining 12 samples were *Legionella*-negative. Therefore, a total of four of 68 samples (5.9%) were positive in the culture with amoebic co-culture.

Ten of the 11 samples positive by culturing were also positive by real-time qPCR without amoebic co-culture (Table 1). The bacterial numbers ranged from 2.6 × 10^4^ (#25) to 1.2 × 10^7^ (#2) cells /L (Table 1). All of the 11 samples positive by culturing were positive by real-time qPCR after amoebic co-culture. The cell numbers ranged from 1.2 × 10^5^ (#25) to 1.1 × 10^12^ (#10) cells /L (Table 1). Using real-time qPCR after amoebic co-culture, more than 10-fold higher bacterial numbers were detected in eight of the 11 *Legionella*-positive samples (Table 1) compared with the same samples without amoebic co-culture.

**Table 1:** Results of the real-time qPCR in the culture-positive 11 bath water samples.

Sample No.	Culture Method			Real-Time qPCR Method (Cell/L)	
	*Legionella* Counts (CFU/L)	*Legionella* Species and Serogroup (SG *^a^*)		Amoebic Co-Culture	Without Amoebic Co-Culture

2	1.6 × 10^5^	*L. pneumophila* SG 3		3.1 × 10^7^	1.2 × 10^7^
4	1.2 × 10^3^	*L. pneumophila* SG 5		3.7 × 10^8^ *	7.3 × 10^4^
10	1.2 × 10^3^	*L. pneumophila* SGs 1, 5		1.1 × 10^12^ *	ND ^b^
25	1.2 × 10^3^	*L. pneumophila* SG 10		1.2 × 10^5^	2.6 × 10^4^
27	7.0 × 10^2^	*L. pneumophila* SG 6		3.2 × 10^6^ *	1.4 × 10^5^
32	3.0 × 10^3^	*L. pneumophila* NT ^c^		6.4 × 10^6^ *	4.8 × 10^4^
41	3.8 × 10^3^	*L. pneumophila* SG10		5.7 × 10^9^ *	9.8 × 10^5^
42 **	2.7 × 10^3^	*L. pneumophila* SGs 1, 3		8.4 × 10^9^ *	2.6 × 10^5^
51 **	7.0 × 10^2^	*L. pneumophila* SG 5		6.1 × 10^7^ *	7.2 × 10^5^
56	8.0 × 10^3^	*L. pneumophila* SGs 1, 5		1.6 × 10^9^ *	7.3 × 10^5^
61	3.2 × 10^3^	*L. pneumophila* SGs 1, 5		7.0 × 10^5^	1.9 × 10^5^

Notes: *: Using real-time qPCR with amoebic co-culture, more than 10-fold higher bacterial numbers compared with the same samples without amoebic co-culture are observed. **: *L. pneumophila* was also detected in the sample by the culture method combined with the amoebic co-culture technique. ^(a)^ SG: serogroup; ^(b)^ ND: not detected (less than 10^2^ cell/L); ^(c)^ NT: non-typable because of non-aggulutinable against polyclonal antisera to *L. pneumophila* serogroup 1 to 14.

### 3.2. Legionella-Negative Culture Samples

Table 2 and Table 3 show the results of the 57 samples that were *Legionella*-negative according to conventional culturing. Of these 57 samples, *Legionella* was detected in 31 samples by real-time qPCR after culturing both with and without amoebae (Table 2). Using real-time qPCR on samples without amoebic co-culture, the bacterial numbers detected ranged from 1.6 × 10^2^ (#15) to 1.4 × 10^5^ (#6) cells/L. However, for samples cultured with amoebic co-culture, real-time qPCR detected bacterial numbers ranging from 1.9 × 10^2^ (#69) to 3.8 × 10^5^ (#19) cells/L (Table 2). In nine of the 31 samples, more than 10-fold higher bacterial numbers were observed compared with the same samples without amoebic co-culture (Table 2). 

Table 3 shows the results of the remaining 26 samples that were *Legionella*-negative by conventional culturing. In these 26 samples, *Legionella* were detected by real-time qPCR in samples co-cultured either with or without amoeba, but not both, or were not detected at all. There were 15 positive samples by real-time qPCR only after amoebic co-culturing (Table 3). The bacterial numbers ranged from 9.1 × 10^2^ (#47) to 1.4 × 10^9^ (#46) cells /L (Table 3). There were five positive samples by real-time qPCR only without amoebic co-culture (#17, #35, #55, #68, and #71). The bacterial numbers ranged from 4.7 × 10^2^ (#35) to 3.1 × 10^3^ (#55) cells /L. Using real-time qPCR with amoebic co-culture techniques, more than 10-fold higher bacterial numbers compared with the same samples without amoebic co-culture were observed in 13 of the 26 culture-negative samples (Table 3).

**Table 2:** Results of the real-time qPCR in the culture-negative 31 bath water samples.

Sample No.	Real-Time qPCR Method (Cell/ L)	
	Amoebic Co-Culture	Without Amoebic Co-Culture
3	5.7 × 10^2^	3.5 × 10^2^
5	5.8 × 10^3^ *	1.8 × 10^2^
6	2.0 × 10^5^	1.4 × 10^5^
7	2.7 × 10^3^	3.0 × 10^3^
15	3.8 × 10^4^ *	1.6 × 10^2^
18	1.9 × 10^3^	2.1 × 10^3^
19	3.8 × 10^5^	7.9 × 10^4^
21	3.4 × 10^3^	2.5 × 10^3^
23	1.9 × 10^3^	3.2 × 10^2^
24	1.0 × 10^4^	8.7 × 10^3^
29	7.8 × 10^4^	1.5 × 10^4^
30	7.2 × 10^2^	4.2 × 10^3^
33	3.8 × 10^4^ *	1.0 × 10^3^
34	7.9 × 10^2^	1.9 × 10^3^
36	2.7 × 10^4^ *	8.0 × 10^2^
38	2.8 × 10^4^	7.0 × 10^3^
39	9.9 × 10^4^ *	4.6 × 10^3^
43	1.9 × 10^4^	3.4 × 10^3^
44	6.1 × 10^4^	2.2 × 10^4^
48	4.4 × 10^2^	5.2 × 10^3^
49	7.8 × 10^3^ *	5.7 × 10^2^
50	1.2 × 10^3^	1.5 × 10^3^
52	9.1 × 10^3^ *	4.7 × 10^2^
53	8.8 × 10^2^	1.0 × 10^4^
54	1.2 × 10^3^	9.7 × 10^2^
57	5.7 × 10^3^	2.3 × 10^3^
59	1.8 × 10^5^	1.0 × 10^5^
63	1.0 × 10^5^ *	1.6 × 10^3^
64	1.1 × 10^5^ *	1.3 × 10^3^
69	1.9 × 10^2^	4.6 × 10^3^
70	1.4 × 10^3^	7.6 × 10^3^

Note: *: Using real-time qPCR with amoebic co-culture, more than 10-fold higher bacterial numbers compared with the same samples without amoebic co-culture are observed.

**Table 3:** Results of the real-time qPCR in the culture-negative 26 samples other than the 31 samples shown in Table 2.

Sample No.	Real-Time qPCR Method (Cell/L)	
	Amoebic Co-Culture	Without Amoebic Co-Culture
11	3.4 × 10^3^ *	ND ^a^
12	2.2 × 10^4^ *	ND
13	3.6 × 10^3^ *	ND
14	1.2 × 10^4^ *	ND
20	9.5 × 10^2^	ND
28	1.2 × 10^4^ *	ND
31	2.1 × 10^4^ *	ND
37	1.0 × 10^4^ *	ND
40	5.3 × 10^3^ *	ND
46	1.4 × 10^9^ *	ND
47	9.1 × 10^2^	ND
58	3.7 × 10^4^ *	ND
62	1.1 × 10^5^ *	ND
65	1.1 × 10^5^ *	ND
66	2.3 × 10^6^ *	ND
17	ND	2.7 × 10^3^
35	ND	4.7 × 10^2^
55	ND	3.1 × 10^3^
68	ND	2.4 × 10^3^
71	ND	7.0 × 10^2^
1	ND	ND
16	ND	ND
22	ND	ND
45	ND	ND
60	ND	ND
67	ND	ND

Notes: *: Using real-time qPCR with amoebic co-culture, more than 10-fold higher bacterial numbers compared with the same samples without amoebic co-culture are observed; ^a^ ND: not detected (less than 10^2^ cells/L).

### 3.3. Comparison of Detection Rates between the Culture and Real-Time qPCR Methods with or without Amoebic Co-Culture Techniques 

Table 4 summarizes the comparison of *Legionella* detection rates between the culture and real-time qPCR methods with or without amoebic co-culture techniques. Positive detection rates of the culture and real-time qPCR methods after with and without amoebic co-culture were 16.2% (11/68), 83.8% (57/68), and 67.6% (46/68), respectively (Table 4). Of the 57 samples that were negative using the culture method, 46 and 36 samples were positive using real-time qPCR after culturing with and without amoebic co-culture, respectively (Table 4). On the other hand, of the 11 samples that were positive using the culture method, zero and one (10.0%) were negative using real-time qPCR with and without amoebic co-culture, respectively. Additionally, of the 21 samples that were negative using real-time qPCR without amoebic co-culture, 10 (48%) were positive when using real-time qPCR after amoebic co-culture. As shown in Table 5, there was a significant difference (chi square = 4.84; *p* = 0.0278) in detection of *Legionella* between real-time qPCR methods with and without the amoebic co-culture techniques.

**Table 4:** Comparison between the culture and real-time qPCR methods with or without amoebic co-culture techniques.

Method	Detection	Real-Time qPCR Method					
		With Amoebic Co-Culture			Without Amoebic Co-Culture		Total
		Positive	Negative		Positive	Negative	
Culture method	positive	11	0		10	1	11
	negative	46	11		36	21	57
Total		57	11		46	22	68

**Table 5:** Relation between amoebic co-culture technique and real-time qPCR method for detection of *Legionella* in bath water samples.

Method	Real-Time qPCR		Total
	Positive	Negative	
With amoebic co-culture	57	11	68
Without amoebic co-culture	46	22	68
Total	99	33	136

Note: Amoebic co-culture technique prior to real-time qPCR results in more sensitive detection of *Legionella* (Chi square = 4.84, *p* < 0.05).

## 4. Discussion

In this study, we applied the amoebic co-culture technique to the detection of *Legionella* from bath water samples, the most common source of legionellosis in Japan. To the best of our knowledge, this is the first report to evaluate the efficacy of the amoebic co-culture technique for detecting *Legionella* from bath water. Co-culturing bath water samples with amoeba did not increase the detection rate of *Legionella*, suggesting that this technique might not be effective for *Legionella* detection in bath water samples. However, improvements to the culture method with amoebic co-culture, such as heating and/or acid treatments for amoebic co-cultured samples and/or shortening the incubation periods with amoeba (3–4 days), may result in more accurate detection of *Legionella* in bath water samples. Further studies are required to test these conditions. 

The application of the amoebic co-culture technique to real-time qPCR detection of *Legionella* from bath water samples increased the bacterial numbers detected and the detection rate of *Legionella* compared with the method without the amoebic co-culture. Real-time qPCR is known to be a much more sensitive method for detecting *Legionella* in environmental water samples compared with the culture method. In previous studies, real-time qPCR was reported to be 1.8–4.0 times more sensitive than the culture method for detecting *Legionella* from water samples collected from a hot water system, a spa, and a cooling tower [15,16,23]. Similarly in our study, real-time qPCR was 4.4 times (44/10 in Table 4) more sensitive than the culture method for detecting *Legionella* from bath water. Furthermore, the amoebic co-culture technique increased the sensitivity of the real-time qPCR and approximately 15 (71.4%) of the 21 previously negative samples tested positive after amoebic co-culture. Our results indicate that application of the amoebic co-culture technique prior to real-time qPCR is more effective for the sensitive detection of *Legionella* from bath water samples.

*L. pneumophila* survives within amoebae by a mechanism similar to one that enables it to survive within macrophages [24,25]. Once taken into amoebae by coiling phagocytosis, *L. pneumophila* escapes the fusion of the phagosome and lysosome and replicates within the amoebae [26]. This ability to invade and multiply within host cells is considered to correlate with its pathogenicity [27]. Segal and Shuman (1999) reported that *L. pneumophila* uses the same genes to multiply within *A. castellanii* and human macrophages [28]. In addition, Cirillo *et al.* (1999) demonstrated that intracellular growth in *A. castellanii* affects monocyte entry mechanisms and enhances the virulence of *L. pneumophila* [29]. Therefore, positive real-time qPCR signals following amoebic co-culture may indicate the existence of viable and virulent *Legionella* in samples [30]. Since real-time qPCR cannot differentiate viable and virulent from non-virulent *Legionella*, the application of an amoebic co-culture technique to real-time qPCR may be useful to detect these virulent *Legionella*. In this study, more than 10-fold higher bacterial numbers were observed by real-time qPCR with the co-culture technique in 30 samples (30/68, 44.1%) compared with the same samples without co-culturing (Tables 1 to 3). This suggests that viable and virulent *Legionella* might be detected. However, higher bacterial numbers were not observed after propagation by amoebae in the remaining 32 samples (excluding six samples, #1, #16, #22, #45, #60, and #67 in Table 3), suggesting that non-virulent and/or non-viable *Legionella* or just DNA might have been detected.

Using the culture method, we detected *Legionella* in 16.2% (11/68) of bath water samples. This result was relatively lower than the *Legionella* detected by Sasahara *et al*. (2004) (49.5%, 52/105), Suzuki *et al*. (2002) (48.0%, 471/981), Karasudani *et al*. (2009) (39.4%, 78/198), and Furuhata *et al*. (2004) (28.7%, 204/710) in Japanese hot springs [7,8,9,10] , and by Lin *et al*. (2007) in Taiwanese hot springs (21.0%, 4/19) [11]. Bath water contaminated with *L. pneumophila* is the main source of legionellosis in Japan. Therefore, surveillance for *Legionella*, particularly *L. pneumophila*, is crucial for legionellosis control. In our study, *Legionella* identified by the culture were *L. pneumophila* SG1, SG3, SG5, SG6, SG10, and NA. Among these *L. pneumophila*, SG1, SG5, and SG6 were the most common serogroups detected from Japanese hot springs and *L. pneumophila* SG1 or SG5 was the causative agent of three large legionellosis outbreaks in Japan [3,4,5]. Thus, routine surveillance for *Legionella* contamination of bath water is essential for preventing legionellosis outbreaks

## 5. Conclusions

The application of the amoebic co-culture technique prior to real-time qPCR was useful for the sensitive detection of *Legionella* from bath water samples. Furthermore, the combination of amoebic co-culture and real-time qPCR might be a useful method to detect viable and virulent *Legionella* because the ability to invade and multiply within FLA is considered to correlate with their pathogenicity for humans.
